# Strategies for selecting/switching chemotherapy and supportive care treatments during COVID‐19 outbreak

**DOI:** 10.1002/cam4.3314

**Published:** 2020-07-20

**Authors:** Quan Li, Leon C. Hwang

**Affiliations:** ^1^ Department of Pharmacy Kaiser Permanente Mid‐Atlantic States Largo MD USA; ^2^ Department of Oncology Mid‐Atlantic PermanenteMedical Group Gaithersburg MD USA

Since the first patient infected with SARS‐CoV‐2 was identified in December 2019, COVID‐19 has spread to 190 countries, resulting in 6 287 771 cases and over 379 941 deaths by June 3rd, 2020.^1^ This public health crisis has threatened the global healthcare system and its operations. Clinical visits and scheduled hospitalizations have to be canceled or postponed. Patients are often advised not to go to the clinic or hospital because of infection risk. Oncology patients are especially at risk of contracting COVID‐19 because they are immunocompromised due to cancer or antitumor treatment. Data has shown that 39%‐54% of cancer patients with COVID‐19 developed severe symptoms, with a mortality rate of up to 28%.^2^, ^3^

Unfortunately, patients receiving active intravenous chemotherapy often have to continue their treatments in clinics or hospitals despite the risk of infection with COVID‐19. While organizations such as ASCO have issued general recommendations for oncology providers, there is limited guidance on how to optimize chemotherapy regimens to reduce the risk of COVID‐19. While selecting or switching regimens is usually based on efficacy, safety, and cost of the regimen; frequency of drug administration and time spent in clinics needs to be taken into heavy consideration during the current health crisis, we discuss here several strategies of selecting or switching chemotherapy and supportive care regimens during the COVID‐19 pandemic (see Table 1).

- Select oral‐based regimens over intravenous drug‐based regimen with the same or similar efficacy. For example, to treat relapsed multiple myeloma, the ixazomib/lenalidomide/dexamethasone (IRd) regimen has the advantage over bortezomib‐based IV regimen in that all medications can be picked up in the outpatient pharmacy, thus, reducing visits to the infusion room.^4^
- Select regimens with similar efficacy but require less frequent visits. For example, 5‐Fluorouracil with oxaliplatin (FOLFOX) requires drug administration every 2 weeks. The capecitabine with oxaliplatin (CAPEOX) regimen, on the other hand, has a similar efficacy but is administered every 3 weeks.^5^ An additional clinical visit is also required for patients treated with FOLFOX to disconnect the 5‐FU pump after the 46‐hour infusion.
- Take “stop‐and‐go” over continuous chemotherapy. For patients with metastatic colorectal cancer treated with the CAPEOX regimen, discontinuation of oxaliplatin is strongly recommended after three months of therapy or sooner until disease progression. This “stop‐and‐go” regimen is non‐inferior to the continuous oxaliplatin‐based regimen.^6^
- Adjust treatment frequencies of the same regimen. Some medications can be given at different intervals. For example, nivolumab and pembrolizumab are considered equally effective in many indications. While many providers usually use nivolumab 240 mg every 2 weeks, it is reasonable to switch to nivolumab 480 mg every 4 weeks during the COVID‐19 outbreak.^7^ There are also data supporting the use of pembrolizumab 400 mg every 6 weeks instead of the approved 200 mg every 3 weeks.^8^ This dosing schedule is approved by the EU and the FDA.
- Switch to different formulations of the same drug, with different pharmacokinetics. This can reduce the frequency and duration of infusion room visits. A good example is oral etoposide, which has a bioavailability of 50%.^9^ A 200 mg/m^2^ oral etoposide can achieve the same blood concentration as an 100 mg/m^2^ intravenous etoposide. As a result, oncologists can reduce the amount of infusion room visits from three days to one day by giving oral etoposide equivalent to the IV etoposide dose on Days 2‐3. Another example is oral mesna, which also has a bioavailability of 50%. Oral mesna can be given to replace IV mesna at 4 hours and 8 hours after ifosfamide administration to shorten the total time required in the infusion room.^10^
- Convert short‐acting to long‐acting medications. Pegfilgrastim/biosimilar products have a much longer half‐life; one dose of pegfilgrastim has the same effect as 7‐10 doses of short‐acting filgrastim/any biosimilar products. When a patient requires multiple daily clinical visits because the patient is incapable of self‐injection, the long‐acting pegfilgrastim and its biosimilar product can be given once per cycle.
- Select the medication requiring single administration over multiple administrations. There are several FDA‐approved IV iron products in the market. While iron sucrose is a preferred agent by many institutions due to the low incidence of hypersensitivity reactions, it requires multiple visits to complete a treatment course. Newer agents such as low molecular weight iron dextran or ferumoxytol allow for a full dose to be given during a single clinical visit, with a similar safety profile.

**Table 1:** Strategies of selecting or switching chemotherapy and supportive care regimens. Strategies for selecting or switching chemotherapy with examples of original and regiments along with recommended dosage and advantages of modifications

Strategies	Examples of Original regimen	Examples of modified regimen	Advantages of modifications
Select oral‐based over intravenous drug‐based regimens	Bortezomib/lenalidomide/dexamethasone Carfilzomib/lenalidomide /dexamethasone	Ixazomib/lenalidomide /dexamethasone	Reduce clinical visit frequency
Select regimens with similar efficacy but require less frequent visits	FOLFOX IV q2w	CAPEOX IV q3w	Reduce clinical visit frequency
Adjust treatment frequencies of the same regimen	Nivolumab 240mg IV q2w Pembrolizumab 200mg IV q3w	Nivolumab 480mg IV q4w Pembrolizumab 400mg IV q6w	Reduce clinical visit frequency
Switch to the same drug with different formulations	Cisplatin IV Day 1 + etoposide IV Days 1‐3 Mesna IV at 0, 4, 8 hours post ifosfamide IV on Days 1‐4 of AIM regimen	Cisplatin IV Day 1 + Etoposide IV Day 1, oral etoposide Days 2‐3 Mesna IV at 0 hour, PO at 4 and 8 hours post ifosfamide IV on Days 1‐4 of AIM regimen	Reduce clinical visit frequency Reduce duration of visits
Convert short‐acting to long‐acting medications	Filgrastim, filgrastim‐sndz subQ Days 2‐7	Pegfilgrastim, pegfilgrastim‐jmdb, pegfilgrastim‐dbqv subQ Day 2 only	Reduce clinical visit frequency
Select the medication requiring single administration over multiple administrations	Iron sucrose IV	Low molecular weight iron dextran IV	Reduce clinical visit frequency
Take “stop‐and‐go” over continuous chemotherapy	CAPEOX q3w	CAPEOX q3w X 3 months followed by capecitabine monotherapy q3w	Reduce clinical visit frequency

Note

q2w: every 2 weeks; q3w: every 3 weeks; q4w: every 4 weeks; q6w: every 6 weeks

While selecting or switching treatment regimens during the COVID‐19 outbreak has advantages of protecting oncology patients, providers should be aware of some potential issues. First, initial patient counseling, staff education, and, a detailed monitoring plan of the “new” regimen are necessary to ensure the success of the treatment and prevent errors. Second, patients may have to cover their co‐pay for oral medication. Finally, reimbursement can be a big challenge for providers when a non‐preferred or a non‐formulary agent is used during the COVID‐19 outbreak. Therefore, it is important to follow guidelines and clinical evidence, and send prior authorization requests early before the treatment starts. It would be optimal for the Centers of Medicare and Medicaid Services (CMS) and private insurance companies to update their policies rapidly to meet emergent practice needs during the COVID‐19 outbreak.

In summary, the COVID‐19 outbreak has created a significant challenge for oncology patients receiving active IV chemotherapy in the clinic. Oncology providers, however, can combine the clinical evidence and pharmacological and pharmacokinetic knowledge of the medications to select new or modify current treatment plans to minimize the risk of viral infection, while still achieving the desired clinical outcome.

## CONFLICT OF INTEREST

The authors have no conflicts of interests to declare.

## AUTHOR CONTRIBUTION

Dr Quan Li contributed to the study design, data analysis, drafting, and final revision of the manuscript. Dr Hwang contributed to the topic idea, editing, and final revision of the manuscript.

## Data Availability

The data sets used of the current study are available from the corresponding author on reasonable request.
